# The low binding affinity of D-serine at the ionotropic glutamate receptor GluD2 can be attributed to the hinge region

**DOI:** 10.1038/srep46145

**Published:** 2017-04-07

**Authors:** Daniel Tapken, Thomas Bielefeldt Steffensen, Rasmus Leth, Lise Baadsgaard Kristensen, Alexander Gerbola, Michael Gajhede, Flemming Steen Jørgensen, Lars Olsen, Jette Sandholm Kastrup

**Affiliations:** 1 Department of Biochemistry I – Receptor Biochemistry, Ruhr University Bochum, Universitätsstraße 150, 44780 Bochum, Germany; 2 Department of Drug Design and Pharmacology, Faculty of Health and Medical Sciences, University of Copenhagen, Jagtvej 162, 2100 Copenhagen, Denmark

## Abstract

Ionotropic glutamate receptors (iGluRs) are responsible for most of the fast excitatory communication between neurons in our brain. The GluD2 receptor is a puzzling member of the iGluR family: It is involved in synaptic plasticity, plays a role in human diseases, *e.g*. ataxia, binds glycine and D-serine with low affinity, yet no ligand has been discovered so far that can activate its ion channel. In this study, we show that the hinge region connecting the two subdomains of the GluD2 ligand-binding domain is responsible for the low affinity of D-serine, by analysing GluD2 mutants with electrophysiology, isothermal titration calorimetry and molecular dynamics calculations. The hinge region is highly variable among iGluRs and fine-tunes gating activity, suggesting that in GluD2 this region has evolved to only respond to micromolar concentrations of D-serine.

Ionotropic glutamate receptors (iGluRs) are present at most neurons in the central nervous system and can be divided into four subfamilies: α-amino-3-hydroxy-5-methylisoxazole-4-propionic acid (AMPA), kainic acid (KA), *N*-methyl-D-aspartic acid (NMDA), and delta receptors^1^. The delta receptor subfamily comprises the two subunits GluD1 and GluD2, which are regarded as iGluRs based solely on sequence identity because direct ligand-gated ion channel function of these receptors has not been observed. However, it was recently reported that the metabotropic glutamate receptor mGlu1 triggers gating of GluD2^2^.

GluD2 is predominantly expressed in the cerebellum on postsynaptic membranes at parallel fiber to Purkinje cell synapses and plays a critical role in long-term depression (LTD) by coordinating interactions between two AMPA receptor phosphorylation sites^3^. In the developing cerebellum, D-serine acts as an endogenous ligand for GluD2 to regulate LTD^4^. Furthermore, *trans*-synaptic neurexin-Cbln1-GluD2 signalling has been shown to induce presynaptic differentiation^5^,^6^,^7^ and synaptic plasticity^7^. These observations indicate that the presence of GluD2 is crucial in the cerebellum. Consequently, several studies have recently reported an important role in human disease, *e.g.* mevalonate kinase deficiency^8^ and cerebellar ataxia and atrophy^9^,^10^,^11^.

The iGluRs are homo- or heterotetrameric receptors, with each of the four subunits consisting of an *N*-terminal domain, a ligand-binding domain (LBD) comprising the two lobes D1 and D2, a membrane-spanning ion channel domain, and an intracellular *C*-terminal domain^12^ (Supplementary Fig. S1). Neutral amino acids such as D-serine and glycine can bind with low affinity (*K*_d_ ~1 mM) to a soluble construct of the GluD2 LBD, causing structural rearrangements in the binding cleft compared to the structure of GluD2 in its *apo* form^13^. Furthermore, D-serine and glycine inhibit spontaneous ion channel conductances in GluD2 containing the *lurcher* mutation (GluD2-Lc; A654T in membrane domain 3) with *IC*_50_ values of 182 μM and 507 μM, respectively^13^. A recent study also reports inhibition of spontaneous currents using another GluD2 mutant and suggests that it is caused by desensitisation^14^.

Furthermore, GluD1 and GluD2 can form functional ligand-gated ion channels when harbouring the LBD of either an AMPA or a kainate receptor, with the GluD2 ion channel pore functioning similarly to those of AMPA and kainate receptors^14^,^15^. Consequently, the key differences between delta receptors and other iGluRs seem to be located within the LBD.

Whereas AMPA and kainate receptors are activated by glutamate binding at the LBD, the NMDA receptors require binding of both glutamate and glycine or D-serine to different subunits of the heteromeric complex for activation^1^. The NMDA receptor subfamily contains three glycine/D-serine-binding subunits: GluN1, GluN3A, and GluN3B. The GluN1 LBD binds D-serine with a *K*_d_ of 7 μM^16^, GluN3A and GluN3B with even higher affinity (*e.g*. GluN3B LBD: *K*_d_ = 163 nM^17^). Thus, the binding affinity of D-serine at GluD2 is more than 100-fold lower than at NMDA receptors. The low binding affinity of D-serine at GluD2 has been a puzzle so far because the binding site residues are similar to those of other iGluRs. In this study, we have investigated this difference in binding affinity as well as potency for D-serine by exchanging GluD2 residues for corresponding residues from GluN1. By two-electrode voltage clamp electrophysiology and isothermal titration calorimetry (ITC) we show that the difference in potency and binding affinity can be attributed to the hinge region connecting the two lobes (D1 and D2) of the GluD2 LBD.

## Results

**Figure 1:**
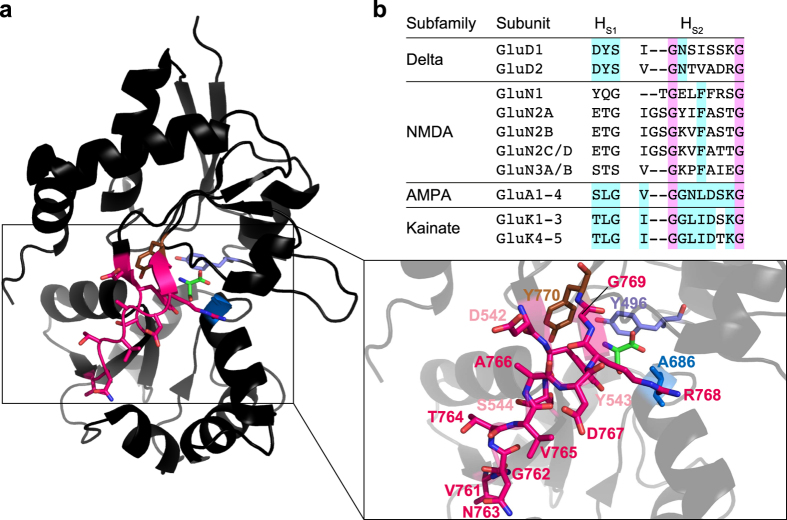
Structure of the GluD2-LBD (PDB ID 2V3U^13^). (**a**) The D1–D2 hinge region (H_S1_ and H_S2_; pink) as well as the positions of point mutations in the binding site generated in this study (Y496F: purple; Y543Q: pink; A686S: blue; Y770F: brown) are indicated. The box on the right shows a magnified view of the D1–D2 hinge region and the D-serine binding site with all residues investigated in this study labelled. (**b**) Comparison of H_S1_ and H_S2_ residues among all 18 iGluR subunits. Residues conserved among all subunits are highlighted in magenta, residues conserved within subfamilies in cyan.

Comparison of amino acid sequences between the LBDs of GluD2 and the glycine/D-serine-binding NMDA receptor subunit GluN1 showed an even distribution of similar residues across the whole LBD. Thus, no specific region in GluD2 was obvious as being responsible for the low binding affinity of D-serine. Therefore, we applied two strategies to investigate the reasons for the low binding affinity: *i*) mutation of GluD2 binding site residues to corresponding residues of GluN1 and *ii*) mutation of the D1–D2 hinge region in the GluD2 LBD to that of GluN1 (Fig. 1).

### Unfavourable enthalpy explains the low binding affinity of D-serine at GluD2

First, we examined the characteristics of D-serine binding to soluble wild-type GluD2-LBD and GluN1-LBD in detail using ITC. In addition to providing binding affinities, this method allows determination of the enthalpic and entropic contributions to binding. We previously found that D-serine binds to GluD2-LBD with a binding affinity (*K*_d_) of 893 μM in an endothermic (Δ*H* = 3 kcal/mol) and thus highly entropy-driven reaction (−*T*Δ*S* = −7 kcal/mol)^13^ (Table 1). By contrast, D-serine bound to the GluN1-LBD with a more than 1000-fold higher affinity of 0.7 μM in an exothermic reaction that is both enthalpy- and entropy-driven (Δ*H* = −3 kcal/mol, −*T*Δ*S* = −5 kcal/mol, Table 1; raw data and isotherms are presented in Supplementary Fig. S2). This indicates that ligand–receptor interactions contribute less favourably to the binding of D-serine at GluD2-LBD than at GluN1-LBD and/or that more strain is present in the GluD2 complex^18^. Thus, an unfavourable enthalpy can explain the low binding affinity of D-serine at GluD2.

### Binding site mutations have moderate effects on binding affinity

To address the hypothesis that the differences in the amino acid residues of the binding site are responsible for the low binding affinity of D-serine at GluD2 compared to GluN1, residues within 4 Å of D-serine were identified for possible mutational analysis based on a structural alignment of the GluD2 LBD (PDB ID 2V3U) and the GluN1 LBD (PDB ID 1PB8). Eleven GluD2 residues were found to be within 4 Å of D-serine: Tyr496, Ala523, Leu524, Thr525, Arg530, Tyr543, Ser685, Ala686, Val687, Trp741, and Asp742. Of these residues, Arg530, Ser685, and Asp742 are conserved in all D-serine-binding iGluR subunits, and Leu524, Thr525, Val687, and Trp741 are conserved between GluD2 and GluN1. Thus, the four residues that are not conserved between GluD2 and GluN1 are: Tyr496, Ala523, Tyr543, and Ala686. Ala523 was excluded from the study because only its backbone carbonyl oxygen atom forms a hydrogen bond to D-serine and its side chain points away from the binding site. Instead, Tyr770, which is located 4.3 Å from D-serine, was included. At the corresponding position, the three D-serine-binding NMDA receptor subunits carry a phenylalanine. Thus, the following point mutations were generated: Y496F, Y543Q, A686S, and Y770F (Fig. 1a, Supplementary Fig. S1).

**Figure 2:**
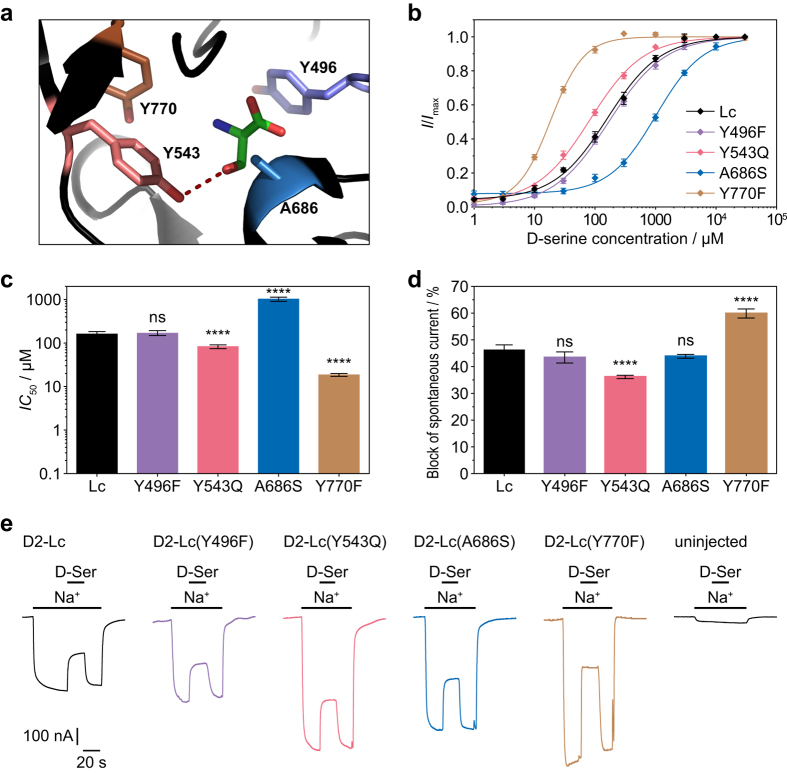
Potencies and efficacies of D-serine at GluD2-Lc binding site mutants. (**a**) Binding site of GluD2 (PDB ID 2V3U^13^) with the four residues mutated in this study shown in stick representation (Y496: purple; Y543: light red; A686: blue; Y770: brown). (**b**) Concentration–response curves for inhibition by D-serine of spontaneous currents mediated by GluD2-Lc and its binding site mutants determined by two-electrode voltage clamp electrophysiology. Each data point is shown as mean ± SEM from 8–11 oocytes. (**c**) Potencies of D-serine at GluD2-Lc and its binding site mutants displayed as *IC*_50_ values calculated from the concentration–response curves shown in *b*. Data are shown as means ± SEM from 8–11 oocytes. The significances of differences from GluD2-Lc were calculated for the log*IC*_50_ values by one-way ANOVA followed by Dunnett’s multiple comparisons test; ns, P > 0.05; ****P ≤ 0.0001. (**d**) Efficacies of D-serine at GluD2-Lc and its binding site mutants, calculated as the fraction of the spontaneous current that is inhibited by D-serine. Data are shown as means ± SEM from 20–30 oocytes. The significances of differences from GluD2-Lc were calculated by one-way ANOVA followed by Dunnett’s multiple comparisons test; ns, P > 0.05; ****P ≤ 0.0001. (**e**) Representative spontaneous currents of GluD2-Lc and its binding site mutants determined by switching from Na^+^-free to Na^+^-containing extracellular solution and their inhibition by D-serine application.

We introduced the four GluD2 binding site mutations one at a time into the GluD2-LBD for ITC experiments (Table 1, raw data and isotherms are presented in Supplementary Fig. S2). The Y496F and Y543Q mutations had only minor effects on D-serine binding affinity (*K*_d_ of 571 μM and 790 μM, Table 1) compared to wild-type GluD2-LBD (809 μM). The interaction of Tyr496 in GluD2 with D-serine does not involve the hydroxyl group of Tyr496, which may explain why the mutation to phenylalanine has little impact. The A686S mutation caused a slight reduction in binding affinity (*K*_d_ = 1090 μM, Table 1). All three D-serine-binding NMDA receptor subunits have a serine at the corresponding position that forms a hydrogen bond with D-serine^16^,^17^. Introducing a serine residue into GluD2 at this position might therefore interfere in an unfavourable manner with the native hydrogen bonding network that involves the hydroxyl group of Tyr543 (Fig. 2a). The Y770F mutation had the largest impact, increasing D-serine affinity 7-fold compared to GluD2-LBD (*K*_d_ = 117 μM *vs*. 809 μM, Table 1). Remarkably, the mutation altered the sign of the enthalpy, making the binding exothermic (Δ*H* = −2 kcal/mol compared to 3 kcal/mol at wild-type GluD2-LBD), but at the same time reduced the favourable entropy (−*T*Δ*S* = −3 kcal/mol compared to −7 kcal/mol at wild-type GluD2-LBD).

### The D1–D2 hinge from GluN1 increases D-serine binding affinity at GluD2-LBD more than 100-fold

The LBD of GluD2, as those of all other iGluRs, has a clamshell-like structure with two lobes, D1 and D2, closing around the ligand when it binds^13^,^19^. We defined the hinge region that changes conformation upon this closure based on comparison of the GluD2-LBD *apo* structure (PDB ID 2V3T) with the structure in complex with the ligand D-serine (PDB ID 2V3U; for details, see Supplementary Fig. S1). The hinge was defined as the following two segments: Asp542-Tyr543-Ser544 (H_S1_) and Val761-Gly762-Asn763-Thr764-Val765-Ala766-Asp767-Arg768-Gly769 (H_S2_) (Fig. 1, Supplementary Fig. S1). The H_S1_ and H_S2_ residues are highly variable among the 18 iGluR subunits, but more conserved within the subfamilies (Fig. 1b).

To analyse the impact of the D1–D2 hinge region on GluD2 function, we replaced the GluD2 hinge residues with the corresponding residues from GluN1 (H_S1_: Tyr-Gln-Gly and H_S2_: Thr-Gly-Glu-Leu-Phe-Phe-Arg-Ser-Gly, Supplementary Fig. S1). We examined the characteristics of D-serine binding to soluble GluD2-LBD-(H)GluN1 in detail using ITC (Table 1, raw data and isotherms are presented in Supplementary Fig. S2). D-serine bound to GluD2-LBD-(H)GluN1 with an affinity (*K*_d_) of 5 μM, corresponding to a 160-fold increase compared to the wild-type GluD2-LBD. Just like at GluN1-LBD, binding to GluD2-LBD-(H)GluN1 was an exothermic, enthalpy- and entropy-driven process with Δ*H* = −4 kcal/mol and −*T*Δ*S* = −3 kcal/mol, contrary to the endothermic process at wild-type GluD2-LBD (Table 1, Supplementary Fig. S2). Thus, the enthalpy change for D-serine binding shifted strongly from unfavourable to favourable, while the entropy change became somewhat less favourable upon transferring the hinge region from GluN1 to the GluD2-LBD. The results therefore suggest that the net change in the number and/or strength of non-covalent interactions from the unbound to the D-serine-bound state is less favourable in GluD2-LBD compared to GluN1-LBD and GluD2-LBD-(H)GluN1 and/or that more strain is introduced in GluD2-LBD.

### The D1–D2 hinge from GluN1 increases D-serine potency at GluD2 *lurcher*

To investigate whether the mutations we had studied on the isolated LBD have an effect on the function of full-length receptors as well, we introduced them into the GluD2 *lurcher* mutant (GluD2-Lc) for electrophysiological analysis by two-electrode voltage clamp in *Xenopus* oocytes (Fig. 2, Table 2). The Y496F and Y543Q mutations had only minor effects on D-serine potency (*IC*_50_ of 158 μM and 81.5 μM, respectively; Fig. 2b,c, Table 2) compared to GluD2-Lc (154 μM), matching the small changes in affinity observed at the LBD construct. The Y543Q mutation resulted in a slightly but significantly decreased D-serine efficacy (36%) compared to GluD2-Lc (46%) (Fig. 2d,e, Table 2). This might be caused by an altered hydrogen bonding network, as the hydroxyl group of Tyr543 in GluD2 forms a hydrogen bond with D-serine^13^ (Fig. 2a). The A686S mutation caused an approximately 6-fold reduction in D-serine potency (1020 μM) (Fig. 2b,c, Table 2), whereas the Y770F mutation had the largest impact, increasing D-serine potency approximately 8-fold compared to GluD2-Lc (*IC*_50_ = 18.5 μM *vs*. 154 μM, Fig. 2b,c, Table 2), a change similar to the change in affinity at the LBD construct.

**Figure 3:**
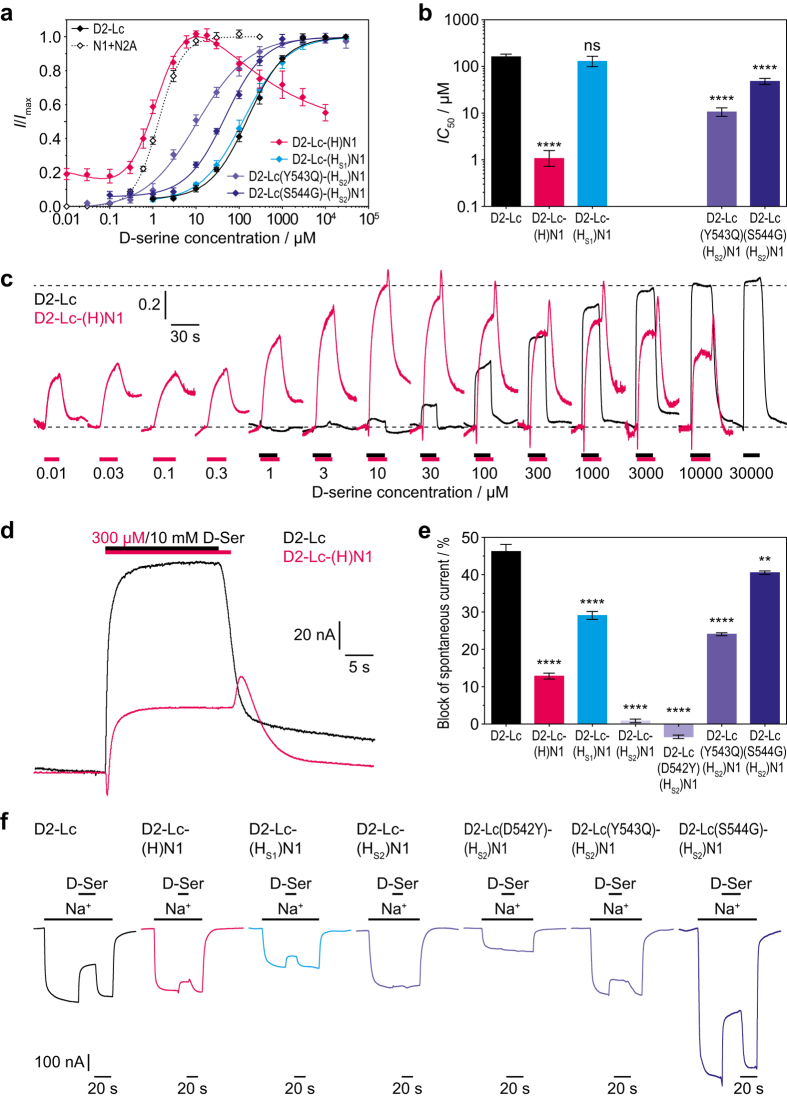
Potencies and efficacies of D-serine at GluD2-Lc D1–D2 hinge region mutants. (**a**) Concentration–response curves for inhibition by D-serine of spontaneous currents mediated by GluD2-Lc, its full hinge region mutant GluD2-Lc(H)GluN1, and three partial hinge region mutants that respond to D-serine. The concentration–response curve for activation of GluN1-1a/GluN2A by D-serine in the presence of 100 μM glutamate is shown for comparison. All curves were determined by two-electrode voltage clamp electrophysiology. Each data point is shown as mean ± SEM from 6–11 oocytes. (**b**) Potencies of D-serine at GluD2-Lc and its full and partial hinge region mutants displayed as *IC*_50_ values calculated from the concentration–response curves shown in *a*. Data are shown as means ± SEM from 6–11 oocytes. The significances of differences from GluD2-Lc were calculated for the log*IC*_50_ values by one-way ANOVA followed by Dunnett’s multiple comparisons test; ns, P > 0.05; ****P ≤ 0.0001. (**c**) Current traces of GluD2-Lc and GluD2-Lc-(H)GluN1 upon application of various D-serine concentrations. The traces are normalized to the amplitude of the current induced by a saturating D-serine concentration (10 mM for GluD2-Lc, 10 μM for GluD2-Lc-(H)GluN1). (**d**) Current traces of GluD2-Lc and GluD2-Lc-(H)GluN1 recorded at high D-serine concentrations. GluD2-Lc-(H)GluN1 shows peak and tail currents upon application and removal of D-serine, respectively. (**e**) Efficacies of D-serine at GluD2-Lc and its full and partial hinge region mutants, calculated as the fraction of the spontaneous current that is inhibited by D-serine. Data are shown as means ± SEM from 20–30 oocytes. The significances of differences from GluD2-Lc were calculated by one-way ANOVA followed by Dunnett’s multiple comparisons test; **P ≤ 0.01; ****P ≤ 0.0001. (**f**) Representative spontaneous currents of GluD2-Lc and its hinge region mutants determined by switching from Na^+^-free to Na^+^-containing extracellular solution and their inhibition by D-serine application.

As observed at the isolated GluD2-LBD, replacing the GluD2 D1–D2 hinge with that of GluN1 had by far the largest impact: D-serine potency at GluD2-Lc increased 130-fold, reducing the *IC*_50_ from 154 μM at GluD2-Lc to 1.2 μM at GluD2-Lc-(H)GluN1, a value virtually identical to the 1.4 μM observed for GluN1-1a (Fig. 3a–c, Table 2). The concentration–response curve of GluD2-Lc-(H)GluN1 was bell-shaped, with maximum inhibition of spontaneous currents occurring at 10 μM D-serine. At D-serine concentrations above the *IC*_50_, we observed peak and tail currents upon application and removal of the agonist, respectively (Fig. 3c,d): When applying D-serine, an inward peak current occurred that added to the spontaneous current of GluD2-Lc. When removing D-serine, an outward tail current was observed that further reduced the spontaneous current beyond the steady-state inhibition by D-serine. Moreover, the maximal, absolute, D-serine-induced steady-state inhibition of spontaneous currents was reduced about 4-fold for the GluD2-Lc-(H)GluN1 chimera (53 ± 8 nA, *n* = 30) compared to GluD2-Lc (222 ± 36 nA, *n* = 20). This reduction was not caused by decreased spontaneous currents, *e.g*. through impaired channel function or reduced expression, as spontaneous currents of GluD2-Lc-(H)GluN1 (385 ± 30 nA, *n* = 30) were not significantly different from those of GluD2-Lc (481 ± 69 nA, *n* = 20). Rather, the D1–D2 hinge region from GluN1 reduced the efficacy of D-serine at GluD2-Lc, as indicated by a reduction of the relative inhibition of spontaneous currents from 46% at GluD2-Lc to 13% at GluD2-Lc-(H)GluN1 (Fig. 3e,f, Table 2).

To further narrow down the GluN1 residues responsible for transferring the high D-serine potency to GluD2, we examined mutants of GluD2-Lc that contained only the GluN1 hinge residues located in S1 (H_S1_) or S2 (H_S2_), respectively. Mutation of the three H_S1_ residues had no significant effect on D-serine potency (*IC*_50_ of 129 μM for GluD2-Lc-(H_S1_)GluN1 *vs*. 154 μM for GluD2-Lc; Fig. 3a,b, Table 2), whereas the relative inhibition of spontaneous currents by D-serine was slightly but significantly reduced from 46% at GluD2-Lc to 29% at GluD2-Lc-(H_S1_)GluN1 (Fig. 3e,f, Table 2). By contrast, introducing the eight H_S2_ residues from GluN1 (GluD2-Lc-(H_S2_)GluN1) almost completely abolished D-serine sensitivity of GluD2-Lc (Fig. 3e,f, Table 2). Spontaneous currents were only slightly reduced from 481 ± 69 nA (*n* = 20) to 302 ± 18 nA (*n* = 20), but they were hardly inhibited by D-serine (0.9 ± 0.4%, *n* = 20, Table 2). Therefore, D-serine potency at this mutant could not be determined. These results show that the H_S1_ and H_S2_ regions need to be replaced simultaneously to confer GluN1-like D-serine potency to GluD2-Lc.

To further verify this conclusion, we analysed three more mutants of GluD2-Lc that contained the H_S2_ region and additionally one of the three H_S1_ residues from GluN1. One of these mutants (GluD2-Lc(D542Y)-(H_S2_)GluN1) remained insensitive to D-serine just like GluD2-Lc-(H_S2_)GluN1, whereas the other two were again inhibited by D-serine (Fig. 3e,f, Table 2). At both of these mutants, D-serine potency was significantly higher than at GluD2-Lc (15-fold for GluD2-Lc(Y543Q)-(H_S2_)GluN1 and 3-fold for GluD2-Lc(S544G)-(H_S2_)GluN1), yet did not come close to the 1.2 μM of GluD2-Lc-(H)GluN1 (Fig. 3a,b, Table 2). This confirms that the full hinge assembly from GluN1 is needed to transfer the high D-serine potency to GluD2.

These findings raised the question whether the opposite is also possible: Can the low D-serine potency of GluD2-Lc be transferred to GluN1 by transferring the hinge ? We analysed the GluN1-1a-(H)GluD2 mutant using electrophysiology in the heteromeric combination with GluN2A and hardly observed any current response to 100 μM glutamate plus 1 mM D-serine (5.8 ± 1.0 nA, *n* = 23). While most oocytes showed currents in the range of the background seen in uninjected control oocytes (2.7 ± 0.2 nA, *n* = 23), a few had slightly higher responses, yet still not sufficient to record a concentration–response relationship.

Because the hinge region from GluN1 had such a strong impact on the GluD2-Lc mutant, we wondered whether it might render the GluD2 wild-type functional. However, this was not the case: GluD2-(H)GluN1 showed barely detectable current responses upon application of 10 μM D-serine (1.1 ± 0.6 nA, *n* = 14) or 1 mM D-serine (2.3 ± 0.4 nA, *n* = 22) that were in the range of currents observed on uninjected control oocytes (0.1 ± 0.1 nA, *n* = 12 at 10 μM D-serine, 2.4 ± 0.2 nA, *n* = 18 at 1 mM D-serine).

### Dynamics of the binding site and hinge region

In order to investigate the structural basis of the changes in binding and receptor function, we performed 100 ns molecular dynamics (MD) simulations on GluD2-LBD, GluN1-LBD, and GluD2-LBD-(H)GluN1 in their *apo* and D-serine-bound states, respectively. All the systems were stable during the simulations, as indicated by the RMSDs on all Cα atoms (Supplementary Fig. S3). The total number of hydrogen bonds from binding site residues to D-serine varied only to a minor extent (Supplementary Fig. S4) as well as D1–D2 domain movements, as indicated by ζ1 and ζ2 values (Supplementary Fig. S5). In addition, D-serine displayed conformations in the receptors that are also observed in solvent, showing that the ligand was not constrained in any unusual conformations (data not shown).

**Figure 4:**
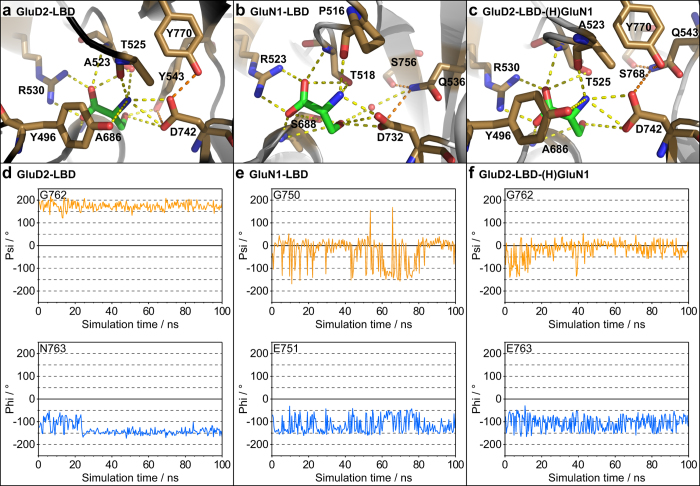
Molecular dynamics simulations on GluD2-LBD, GluN1-LBD, and GluD2-LBD-(H)GluN1 in their D-serine-bound forms. (**a**) Hydrogen bonds (yellow dashed lines) between D-serine (green) and binding site residues (sand) as well as from Asp742 to Tyr543 and Tyr770 (orange dashed lines) in GluD2-LBD. All hydrogen bonds to D-serine observed during the simulation have been mapped onto the X-ray structure (PDB ID 2V3U). (**b**) Hydrogen bonds between D-serine and binding site residues as well as from Gln536 to Asp732 and Ser756 (orange dashed lines) in GluN1-LBD. All hydrogen bonds to D-serine observed during the simulation have been mapped onto the X-ray structure (PDB ID 1PB8). (**c**) Hydrogen bonds between D-serine and binding site residues as well as from Gln543 to Asp742, Ser768, and Tyr770 in GluD2-LBD-(H)GluN1. All hydrogen bonds to D-serine observed during the simulation have been mapped onto the model structure. (**d–f**) Variation of the phi and psi torsional angles for two residues in the H_S2_ region during the 100 ns MD simulation. (**d**) GluD2-LBD with D-serine. (**e**) GluN1-LBD with D-serine. (**f**) GluD2-LBD-(H)GluN1 with D-serine. For phi and psi values of all the residues in H_S2_, see Supplementary Fig. S7.

During the MD simulations, a total of seven amino acid residues in GluD2-LBD (Tyr496, Ala523, Thr525, Arg530, Tyr543, Ala686, Asp742) and five residues in GluN1-LBD (Pro516, Thr518, Arg523, Ser688, Asp732) as well as water molecules were engaged in hydrogen bonding to D-serine (Supplementary Table S1, water molecules not included). Overall, the hydrogen bonding patterns from D-serine to binding site residues are similar in GluD2 and GluN1, supporting the observation that point mutations of binding site residues affect binding affinity and potency of D-serine only to a minor extent. The most pronounced differences arise from: *i*) the presence of Tyr496 in GluD2-LBD (Phe484 in GluN1-LBD) whose hydroxyl group forms hydrogen bonds to the ammonium group of D-serine, *ii*) Tyr543 in GluD2-LBD and Gln536 in GluN1-LBD, which form a direct and a water-mediated hydrogen bond to the hydroxyl group of D-serine, respectively, and *iii*) Ser688 in GluN1-LBD (Ala686 in GluD2-LBD) whose hydroxyl group is involved in hydrogen bonding to both the carboxylate and hydroxyl group of D-serine (Fig. 4a,b).

To address the effects of introducing the D1–D2 hinge region from GluN1 into GluD2, we transferred these residues into the GluD2-LBD *apo* and D-serine-bound structures and then performed 100 ns MD simulations. First, we analysed the overall flexibility of the six structures. As expected, the *apo* structures were generally more flexible than the D-serine-bound structures (Supplementary Figs S3 and S5). Whereas flexibility was more pronounced in the hinge regions in GluN1-LBD and GluD2-LBD-(H)GluN1, the long H_S2_ hinge region in GluD2-LBD underwent a conformational change after 40 ns (Supplementary Figs S6 and S7). Subsequently, we analysed the fluctuations in backbone phi and psi angles of each H_S2_ residue (Supplementary Fig. S7). Large fluctuations were seen in the psi angle of Gly750 in GluN1-LBD and the corresponding Gly762 in GluD2-LBD-(H)GluN1 as well as in the phi angle of Glu751 in GluN1-LBD and Glu763 in GluD2-LBD-(H)GluN1 (Fig. 4e,f). On the contrary, the corresponding phi and psi angles seemed stable in GluD2-LBD (Fig. 4d). This indicates more backbone flexibility in the H_S2_ hinge region in GluN1-LBD and GluD2-LBD-(H)GluN1 than in GluD2-LBD and points towards structural differences between the hinge regions in GluD2 and GluN1 that might be of importance for determining binding affinity of D-serine.

## Discussion

In summary, this study defines the D1–D2 hinge region as being responsible for the low binding affinity of D-serine at the ligand-binding domain of GluD2 and low potency of D-serine at the GluD2 *lurcher* mutant. By contrast, changing binding site residues by single point mutations had only minor effects on the binding affinity of D-serine at GluD2.

We found that replacing the hinge region that connects the two lobes of the LBD with that from the NMDA receptor subunit GluN1 increased affinity of D-serine at a soluble GluD2-LBD construct as well as potency of D-serine at the spontaneously open GluD2 *lurcher* mutant more than 100-fold, bringing both values close to those at GluN1. Whereas D-serine binding is endothermic and entropy-driven at the wild-type GluD2-LBD, the hinge region from GluN1 rendered it exothermic, so it became both enthalpy- and entropy-driven, as observed at GluN1. Furthermore, molecular dynamics simulations suggested that the conformation of the GluN1 H_S2_ hinge region is preserved when transferring it to the GluD2 LBD. The reverse experiment, replacing the hinge region of GluN1 with that from GluD2, produced a non-functional chimera, so D-serine potency could not be determined. Similar observations of non- or barely functional chimeras composed of regions or domains that are perfectly functional in the wild-type protein have previously been made^20^,^21^.

At the GluD2 *lurcher* mutant, introducing the hinge region from GluN1 not only changed D-serine potency but also other properties of the receptor. Application of high D-serine concentrations induced a transient inward peak current followed by a reduced inhibition of the spontaneous current compared to GluD2-Lc. As the inhibition of spontaneous currents is likely caused by desensitisation^13^, these findings suggest that the GluN1 hinge slows and reduces desensitisation at GluD2-Lc, so an activation peak can be observed before a less pronounced desensitisation sets in. Another striking feature of GluD2-Lc-(H)GluN1 is its bell-shaped concentration–response curve, with a maximal inhibition of spontaneous currents at 10 μM D-serine and smaller steady-state inhibition followed by apparently outward tail currents upon D-serine washout at higher concentrations. The tail currents can probably be explained by lower D-serine concentrations briefly reached during washout that induce maximal inhibition. Bell-shaped concentration–response curves have been reported for other glutamate receptors, *e.g*. in heteromeric assemblies such as GluN1/GluN3^22^ and GluK2/GluK5^23^,^24^ that contain binding sites with different agonist affinities on subunits with different gating properties or in complexes with auxiliary proteins such as TARPs that can dissociate from the receptor upon agonist binding^25^. Investigating the mechanistic reasons for the unusual concentration-dependent behaviour of the homomeric GluD2-Lc-(H)GluN1 will require extensive analysis of its gating properties.

In GluD2-Lc, the introduction of the H_S1_ part of the hinge from GluN1 (Tyr-Gln-Gly) alone did not have any effect on D-serine potency, whereas the long H_S2_ part alone completely abolished D-serine efficacy (Table 2). This illustrates that the full hinge assembly from GluN1 is needed to transfer the high D-serine potency to GluD2-Lc. The single most important residue in H_S1_ for increasing D-serine potency seems to be at position 543 in GluD2, where introduction of Gln instead of Tyr (GluD2-Lc(Y543Q)-(H_S2_)GluN1) leads to a 15-fold increased potency in conjunction with the H_S2_ residues from GluN1. The corresponding Gln536 in GluN1-LBD was observed to form hydrogen bonding contacts to the important D-serine-interacting residue Asp732 and the H_S2_ hinge region residue Ser756 (Fig. 4b). Similarly, in GluD2-LBD-(H)GluN1 major contacts are seen from the new H_S1_ hinge residue Gln543 to the new H_S2_ hinge residue Ser768 during the MD simulation, as well as to Asp742 and Tyr770 (Fig. 4c). Introduction of Tyr instead of Asp542 (GluD2-Lc(D542Y)-(H_S2_)GluN1) did not rescue D-serine efficacy.

The hinge region is highly variable among iGluR subfamilies (Fig. 1b) and fine-tunes gating activity. For example, in NMDA receptors, the H_S1_ hinge residue Tyr535 in GluN1 (corresponding to Asp542 in GluD2) has been shown to control the deactivation rate of heteromeric GluN1/GluN2A receptors: mutations increasing hydrophobic contacts slowed deactivation, whereas mutations decreasing hydrophobic contacts accelerated it^26^,^27^. Therefore, it is tempting to speculate that the D1–D2 hinge region in GluD2 has evolved in a way only allowing the receptor to respond to micromolar concentrations of D-serine. It means that D-serine/glycine can dissociate rapidly from the receptor to terminate a response. D-serine has previously been shown to regulate LTD at synapses between parallel fibers and Purkinje cells in the immature cerebellum through its action as an endogenous ligand for GluD2^7^. The inhibitory effect of D-serine on the parallel fiber EPSCs was shown to increase in a dose-dependent manner with an apparent *EC*_*50*_ of 147 μM. This *EC*_*50*_ is in agreement with what we observe at GluD2-Lc (154 μM). However, although D-serine binding to GluD2 has been demonstrated to play an important physiological role in regulating cerebellar LTD and motor coordination^4^, direct ligand-gated ion channel function of GluD2 has not been observed yet. Therefore, the specific role of the hinge region for the physiological function of GluD2 remains elusive until the molecular mechanism of GluD2 signalling is fully understood.

In conclusion, the present study opens new perspectives for studies of the function of GluD2 and the unique characteristics of the D1–D2 hinge region. Future comparative studies of differences in this hinge region among the 18 iGluR subunits would likely provide essential information of general interest on these receptors.

## Methods

### GluD2-LBD constructs

The constructs for expression of wild-type rat GluD2-LBD^13^ and GluD2-LBD mutants are composed of an N-terminal extension coding for a His-tag and a trypsin cleavage site, the GluD2 S1 residues Gly440–Arg551, a Gly-Thr linker, and GluD2 S2 residues Ser664–Leu813. Amino acid residues are numbered based on the full-length polypeptide sequence (GenBank accession no.: NM_024379) including the signal peptide of 23 residues. Mutations in GluD2-LBD were introduced using overlap extension PCR^28^, except for the GluD2-LBD-(H)GluN1 mutant, which was produced by GenScript USA Inc. (Piscataway, NJ, USA).

### GluN1-LBD construct

The GluN1-LBD construct comprises an N-terminal region coding for a His-tag and a thrombin cleavage site, the GluN1 S1 residues Met394-Lys544, a Gly-Thr linker, and GluN1 S2 residues Arg663-Ser800^16^. Amino acid residues are numbered based on the full-length polypeptide sequence including the signal peptide (UniProt accession no. P35439).

### Expression and purification

Wild-type GluD2-LBD and mutants were expressed in the *E. coli* cell line Origami 2 (Novagen, Madison, WI, USA) using the T7 expression vector pET28+ as previously described^13^. In brief, the protein was purified by affinity chromatography using a HisTrap FF column (GE Healthcare, Hillerød, Denmark). The His-tag was cleaved by trypsin digestion, leaving one N-terminal remnant Gly. The final purification step comprised ion exchange chromatography using a HiTrap FF column (GE Healthcare). The eluted protein was then dialysed three times against ITC buffer (100 mM HEPES, 100 mM NaCl, 2 mM KCl, 10% glycerol, pH 7.0).

GluN1-LBD was expressed in *E. coli* Origami B (DE3) (Novagen) using the T7 expression vector pET22c as previously described^29^. Expression was performed in LB medium overnight at 20 °C by IPTG induction (0.1 mM). In brief, the protein was purified by affinity chromatography using a HisTrap FF column (GE Healthcare). The His-tag was cleaved overnight with thrombin (2 U/mg protein) at room temperature, which leaves one remnant N-terminal Gly. As a final purification step, the protein was purified by ion exchange chromatography using a HiTrap SP FF column (GE Healthcare). The protein buffer was exchanged to ITC buffer using dialysis (three times buffer exchange).

All proteins were checked for correct folding using circular dichroism (CD) spectroscopy (data not shown). In addition, size-exclusion chromatography confirmed that GluD2-LBD, GluN1-LBD and GluD2-LBD-(H)-GluN1 elute in the same volume, indicating that all proteins are monomeric in solution (Supplementary Fig. S8).

### GluD2 *lurcher* constructs

All cDNAs for expression in *Xenopus* oocytes were cloned into the vector pSGEM^30^. Point mutants of GluD2-Lc were constructed by overlap extension PCR with mutagenic oligonucleotide primers^28^. The GluD2-Lc-(H)GluN1 chimera was constructed by transferring the S1- and S2-encoding sequences from GluD2-LBD-(H)GluN1 to GluD2-Lc via overlap extension PCR using tail primers. The S1 and S2 sequences were transferred separately, yielding GluD2-Lc-(H_S1_)GluN1 and GluD2-Lc-(H_S2_)GluN1 constructs. GluD2-Lc-(H)GluN1 was then produced from these by another overlap extension PCR. Complementary RNA (cRNA) for injection into oocytes was synthesised from 1 μg of linearised template DNA with the mMESSAGE mMACHINE *in vitro* transcription kit (Ambion, Austin, TX, USA).

### Isothermal titration calorimetry

The concentration of the proteins in ITC buffer (100 mM HEPES, 100 mM NaCl, 2 mM KCl, 10% glycerol, pH 7.0) was between 0.02 mM and 0.09 mM as determined by UV absorption. D-serine was dissolved in ITC buffer that had been used for the final round of protein dialysis to a concentration of 0.2 mM–21 mM, and afterwards filtered through a 0.22 μm filter. The ITC experiments were carried out on either a VP-ITC calorimeter (MicroCal, GE Healthcare) or an ITC200 calorimeter (MicroCal, GE Healthcare) with cell volumes of 1.4 mL and 200 μL, respectively. Experiments were performed at 25 °C for GluD2-LBD and GluD2-LBD binding site mutants and at 20 °C for GluN1-LBD and GluD2-LBD-(H)GluN1. The experiments were performed as 20 injections of D-serine into the protein solution. At the VP-ITC calorimeter the injection volume was 2 μL for the first and 15 μL for all following injections, at the ITC200 calorimeter it was 0.2 μL for the first and 2 μL for all following injections. The data were analysed using Origin 7.0 (OriginLabs, Northampton, MA, USA) using a single binding site model^31^. The baseline was manually corrected for a few data sets and the first data point discarded.

### Electrophysiology on *Xenopus laevis* oocytes

Oocytes were obtained by surgically removing parts of the ovaries from *Xenopus laevis* (Nasco, Fort Atkinson, WI, USA) anaesthetised with 1.5 g/L 3-aminobenzoic acid ethylester (Sigma, Taufkirchen, Germany). The ovary clippings were digested with 784 U/mL (4 mg/mL) collagenase type I (Worthington, Lakewood, NJ, USA) in Ca^2+^-free Barth’s solution (88 mM NaCl, 1.1 mM KCl, 2.4 mM NaHCO_3_, 0.8 mM MgSO_4_, 15 mM HEPES-NaOH, pH 7.6) with gentle agitation for 1.5–2 h at 20 °C to remove the follicular cell layer. After stopping the collagenase digestion by washing with Barth’s solution (88 mM NaCl, 1.1 mM KCl, 2.4 mM NaHCO_3_, 0.3 mM Ca(NO_3_)_2_, 0.4 mM CaCl_2_, 0.8 mM MgSO_4_, 15 mM HEPES-NaOH, pH 7.6), defolliculated oocytes of stages V and VI were selected and maintained at 16 °C in Barth’s solution supplemented with 100 μg/mL gentamicin, 40 μg/mL streptomycin, and 63 μg/mL penicillin. Oocytes were injected with 11 ng cRNA (10 ng of each cRNA for co-expression of GluN1 and GluN2A) using a nanoliter injector (WPI, Sarasota, FL, USA). Four days after cRNA injection, current responses were recorded under voltage clamp at –70 mV with a Turbo Tec-10CD amplifier (npi electronic, Tamm, Germany) controlled by Patchmaster software (HEKA, Lambrecht, Germany). Currents were filtered with a 20 Hz low-pass filter and then digitised with a sampling rate of 50 Hz. Recording electrodes were pulled from borosilicate glass (Hilgenberg, Malsfeld, Germany) with an L/M-3P-A vertical pipette puller (List-Medical), filled with 3 M KCl, and had resistances of 0.5–5 MΩ (voltage electrode) or 0.5–1.5 MΩ (current electrode). Recordings were performed in a 50 μL chamber under constant superfusion with extracellular solution at a flow rate of 3–5 mL/min. The standard extracellular solution was Ca^2+^- and Mg^2+^-free barium normal frog Ringer’s solution (Ba-NFR; 115 mM NaCl, 2.5 mM KCl, 1.8 mM BaCl_2_, 10 mM HEPES-NaOH, pH 7.2). D-serine potencies were determined by measuring steady-state current responses induced by the application of increasing D-serine concentrations to the same oocyte. The obtained responses were normalised to the maximal response induced by a saturating D-serine concentration and the normalised data from six to eleven oocytes averaged and fitted to the Hill equation using Prism (GraphPad, San Diego, CA, USA). D-serine efficacies were investigated by determining the fraction of the spontaneous current through GluD2-Lc channels that was inhibited by a saturating concentration of D-serine. To this end, the amplitude of the spontaneous current was determined by switching from a Na^+^-free solution containing the impermeable cation NMDG (NMDGR; 115 mM NMDG-Cl, 2.5 mM KCl, 1.8 mM BaCl_2_, 10 mM HEPES, pH 7.2) to Na^+^-containing Ba-NFR. Then D-serine in Ba-NFR was applied and the inhibition of the spontaneous current measured. Finally, Ba-NFR and then NMDGR were applied again to check for reversibility.

### Preparation of structures for molecular dynamics simulations

Wild-type GluD2-LBD, GluD2-LBD-(H)GluN1, and wild-type GluN1-LBD were prepared as *apo* structures and with D-serine bound as described below.

For calculations on the wild-type GluD2 *apo* structure, the structure of *apo* GluD2-LBD (PDB ID 2V3T, molB) was used. The hinge region residues 763–764 are not present in the structure. These residues were built according to the weak electron density. Six C-terminal residues were adopted from molA. All water molecules within 5 Å of the protein were kept.

For wild-type GluD2 with D-serine bound, the structure of GluD2-LBD with D-serine (PDB ID 2V3U) was used. As loop 715–719 was not built in this structure, the loop from the GluD2-LBD *apo* structure (PDB ID 2V3T, molB, residues 713–719) was modelled in. Furthermore, hinge region residues 763–765 were not present in the structure. These residues were built according to the weak electron density. D-serine and all water molecules within 5 Å of the protein were kept.

For wild-type *apo* GluN1, the structure of *apo* GluN1-LBD (PDB ID 4KCC) was used. Loops 441–448 and 491–495 are missing in the structure and were adopted from the structure of GluN1-LBD with ACPC (PDB ID 1Y20, 440–449 and 490–496, respectively). Residues built as Ala were changed to corresponding residues in the protein sequence. Side-chain rotamers that did not provide clash with the protein and occur frequently were chosen. All water molecules within 5 Å of the protein were kept.

For wild-type GluN1 with D-serine bound, the structure of GluN1-LBD with D-serine (PDB ID 1PB8) was used. Loop 441–447 was missing in the structure and was adopted from the structure of GluN1-LBD with ACPC (PDB ID 1Y20, 440–448). Residues built as Ala were changed to corresponding residues in the protein sequence. Side-chain rotamers that did not provide clash with the protein and occur frequently were chosen. D-serine and all water molecules within 5 Å of the protein were kept.

For constructing the *apo* GluD2-LBD-(H)GluN1 mutant, GluN1 residues Tyr535, Gln536, and Gly537 were inserted instead of GluD2 residues Asp542, Tyr543, and Ser544 (H_S1_) and GluN1 residues Thr749, Gly750, Glu751, Leu752, Phe753, Phe754, Arg755, Ser756, and Gly757 instead of GluD2 residues Val761, Gly762, Asn763, Thr764, Val765, Ala766, Asp767, Arg768, and Gly769 (H_S2_). The side chain conformation of seven residues was adjusted to avoid clash with surrounding residues. One water molecule in the vicinity of the D1–D2 hinge region that would cause steric clash was deleted.

For constructing the GluD2-LBD-(H)GluN1 mutant with D-serine bound, GluN1 residues Tyr535, Gln536, and Gly537 were inserted instead of GluD2 residues Asp542, Tyr543, and Ser544 (H_S1_) and GluN1 residues Thr749, Gly750, Glu751, Leu752, Phe753, Phe754, Arg755, Ser756, and Gly757 instead of GluD2 residues Val761, Gly762, Asn763, Thr764, Val765, Ala766, Asp767, Arg768, and Gly769 (H_S2_). Eight water molecules in the vicinity of the D1–D2 hinge region that would cause steric clash were deleted.

### Molecular dynamics simulations

The Schrödinger Software Release 2013–2 (Schrödinger, LLC, New York, 2013) and the Protein Preparation Wizard 2013-2 (Epik version 2.4, Impact version 5.9, Prime version 3.2, Schrödinger, LLC, New York, 2013)^32^ were used to prepare protein structures for the MD simulations. All crystallographic waters were kept during the preprocessing step. Missing side chains were added, selenomethionine converted to methionine and buffer molecules deleted. The hydrogen bond assignment was done using default settings. The tleap program of the Amber 12 package (Case DA, Darden TA, Cheatham III TE, Simmerling CL, Wang J, Duke RE, Luo R, Walker RC, Zhang W, Merz KM, Roberts B, Hayik S, Roitberg A, Seabra G, Swails J, Götz AW, Kolossváry I, Wong KF, Paesani F, Vanicek J, Wolf RM, Liu J, Wu X, Brozell SR, Steinbrecher T, Gohlke H, Cai Q, Ye X, Wang J, Hsieh M-J, Cui G, Roe DR, Mathews DH, Seetin MG, Salomon-Ferrer R, Sagui C, Babin V, Luchko Gusarov TS, Kovalenko A & Kollman PA (2012), AMBER 12, University of California, San Francisco) was used to create prmtop and prmcrd, topology and coordinate files of the proteins, respectively, with the ff99SB force field^33^. The proteins were neutralised with counter ions. The tool ACPYPE^34^,^35^,^36^ was used to convert prmtop and prmcrd to GROMACS format, and GROMACS 5.0.2^37^ was used for periodic box generation, solvation, equilibration and MD simulations (Abraham MJ, van der Spoel D, Lindahl E & Hess B (2014) The GROMACS development team, GROMACS User Manual Version 5.0.2). All protein structures were solvated in a truncated octahedron box with TIP3P water. Equilibration of the systems included minimisation of the solvent, gradual heating from 0 to 300 K in the NVT ensemble and 1 ns simulation at 300 K and 1.01325 bar in the NPT ensemble. Finally, production MD runs of 100 ns were carried out. The MD simulations were analysed with VMD 1.9.1^38^.

## Additional Information

**How to cite this article:** Tapken, D. *et al*. The low binding affinity of D-serine at the ionotropic glutamate receptor GluD2 can be attributed to the hinge region. *Sci. Rep.* **7**, 46145; doi: 10.1038/srep46145 (2017).

**Publisher's note:** Springer Nature remains neutral with regard to jurisdictional claims in published maps and institutional affiliations.

## Supplementary Material

Supplementary Information

## Figures and Tables

**Table 1:** Thermodynamics of D-serine binding to wild-type GluD2-LBD, wild-type GluN1-LBD, the GluD2-LBD-(H)GluN1 hinge region mutant as well as GluD2-LBD binding site mutants determined by isothermal titration calorimetry.

Protein	*K*_d_ (μM)	Δ*H* (kcal/mol)	−*Τ*Δ*S* (kcal/mol)	*N*	*n*^a^
***T***** = 20 °C**					
GluD2-LBD^b^	893	3.1	−7.2	1^c^	
GluN1-LBD	0.7 ± 0.3^d^	−3.2 ± 0.8	−5.0 ± 1.2	0.33 ± 0.02	3
GluD2-LBD-(H)GluN1	5.4 ± 1.7	−4.3 ± 0.6	−2.7 ± 0.8	0.79 ± 0.10	3
***T***** = 25 °C**					
GluD2-LBD	809 ± 4	3.1 ± 0.2	−7.13 ± 0.05	1^c^	3
GluD2-LBD(Y496F)	571 ± 115	1.4 ± 0.3	−5.8 ± 0.3	1^c^	3
GluD2-LBD(Y543Q)	790 ± 112	0.71 ± 0.06	−5.00 ± 0.05	1^c^	3
GluD2-LBD(A686S)	1090 ± 231	1.1 ± 0.1	−5.11 ± 0.02	1^c^	3
GluD2-LBD(Y770F)	117 ± 11	−2.3 ± 0.1	−2.8 ± 0.3	1^c^	3

^a^*n* = number of experiments.

^b^Data from Naur *et al*.^13^.

^c^The stoichiometry *(N)* was fixed at 1.

^d^Standard deviation given.

**Table 2:** D-serine potencies (*IC*_50_ or *EC*_50_) and efficacies (%) at GluD2-Lc D1–D2 hinge region and binding site mutants determined by two-electrode voltage clamp electrophysiology in *Xenopus* oocytes.

Protein	*IC*_50_ (μM) [*EC*_50_ (μM) for GluN1/GluN2A]		*n*	D-serine efficacy (%)	*n*
	Mean	95% confidence interval		Mean ± SEM	
GluD2-Lc	154	138–173	11	46.2 ± 1.9	30
GluN1-1a (with GluN2A)	1.35	1.26–1.45	8	n/a*	
GluD2-Lc-(H)GluN1	1.19	0.84–1.69	6	12.9 ± 0.8	30
GluD2-Lc-(H_S1_)GluN1	129	100–165	9	29.1 ± 1.1	20
GluD2-Lc-(H_S2_)GluN1	n/a**	n/a**		0.9 ± 0.4	20
GluD2-Lc(D542Y)-(H_S2_)GluN1	n/a**	n/a**		–3.4 ± 0.5	20
GluD2-Lc(Y543Q)-(H_S2_)GluN1	10.5	8.5–13.0	8	24.1 ± 0.4	20
GluD2-Lc(S544G)-(H_S2_)GluN1	47.9	41.4–55.6	8	40.6 ± 0.5	20
GluD2-Lc(Y496F)	158	141–176	11	43.5 ± 2.0	20
GluD2-Lc(Y543Q)	81.5	73.4–90.4	11	36.2 ± 0.6	20
GluD2-Lc(A686S)	1020	911–1143	8	43.9 ± 0.7	20
GluD2-Lc(Y770F)	18.5	17.1–20.0	8	59.9 ± 1.7	20

*Efficacy cannot be calculated for an excitatory response.

^**^No response to D-serine.
