# Supplementary material for: The low binding affinity of D-serine at the ionotropic glutamate receptor GluD2 can be attributed to the hinge region
Source: Sci Rep. 2017 Apr 7;7:46145. doi: 10.1038/srep46145 (PMC5384001; doi:10.1038/srep46145)
Supplement: Supplementary Information [file srep46145-s1.pdf]

Supporting Information

The low binding affinity of D-serine at the ionotropic glutamate receptor GluD2 can be attributed to the hinge region

Daniel Tapken[1,3], Thomas Bielefeldt Steffensen[1,2,3], Rasmus Leth[2], Lise Baadsgaard Kristensen[2], Alexander Gerbola[2], Michael Gajhede[2], Flemming Steen Jørgensen[2], Lars Olsen[2], and Jette Sandholm Kastrup[2*]

[1]Department of Biochemistry I – Receptor Biochemistry, Ruhr University Bochum, Universitätsstraße 150, 44780 Bochum, Germany. [2]Department of Drug Design and Pharmacology, Faculty of Health and Medical Sciences, University of Copenhagen, Jagtvej 162, 2100 Copenhagen, Denmark. [3]These authors contributed equally to the work.

*Correspondence should be addressed to J.S.K. (jsk@sund.ku.dk) or D.T. (electrophysiology: daniel.tapken@rub.de) or L.O. (ITC and computational studies: lo@sund.ku.dk).

Table of Contents

Figure S1: Topology of iGluRs, sequence of GluD2, and definition of the D1–D2 hinge region of the GluD2 LBD.

Figure S2: Isothermal titration calorimetry data on binding of D-serine to wild-type GluN1-LBD (**a**), wild-type GluD2-LBD (**b**), binding site mutants (**c–f**), and GluD2-LBD-(H)GluN1 (**g**).

Figure S3: RMSD on all Cα atoms during the 100 ns MD simulation.

Figure S4: Total number of hydrogen bonds from binding site residues to D-serine during the 100 ns MD simulation as calculated with GROMACS (gmx hbond).

Figure S5: ζ1 and ζ2 values during the 100 ns MD simulations as calculated with GROMACS (gmx hbond).

Figure S6: Structures colored according to flexibility during 100 ns MD simulation.

Figure S7: Variation of the phi and psi torsional angles for the H_{S2} region during the 100 ns MD simulation.

Figure S8: Size-exclusion chromatography on GluD2-LBD, GluN1-LBD, and GluD2-LBD-(H)GluN1.

Table S1. Hydrogen bonding between D-serine and binding site residues during the 100 ns MD simulation.

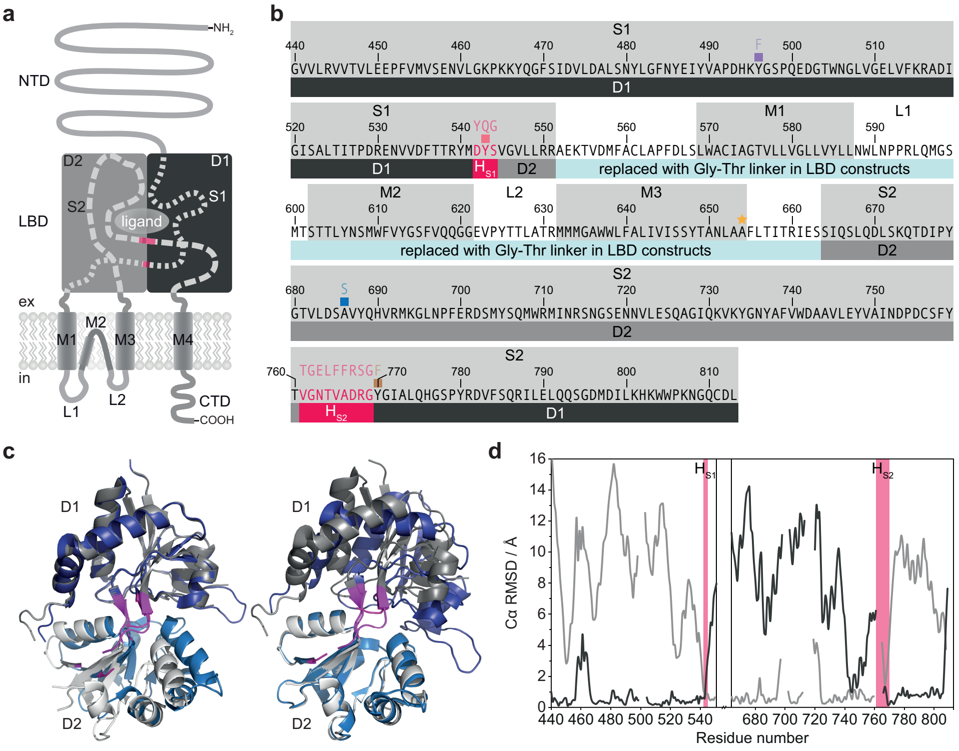

Fig. S1. Topology of iGluRs, sequence of GluD2, and definition of the D1–D2 hinge region of the GluD2 LBD. (**a**) Domain structure and topology of an iGluR subunit. The LBD is composed of the two sequence stretches S1 and S2 and fold into two lobes, D1 and D2, each containing residues from S1 as well as S2. (**b**) Sequence of the GluD2 LBD and the transmembrane regions connecting S1 and S2. The D1–D2 hinge region is highlighted in pink and the binding site residues mutated in this study are indicated by squares. Letters above the sequence denote the corresponding residues in GluN1. The star indicates the position of the A-to-T *lurcher* mutation. (**c**) Superimposition of the GluD2-LBD *apo* structure (PDB ID 2V3T, D1 dark grey, D2 light grey) with the structure in complex with the ligand D-serine (PDB ID 2V3U, D1 dark blue, D2 light blue). The structures were aligned on either the D1 residues (left) or the D2 residues (right). The D1–D2 hinge region as determined from the diagram shown in *d* is highlighted in pink. (**d**) Root-mean-square deviations (RMSD) between Cα atoms of the GluD2-LBD *apo* structure and GluD2-LBD in complex with D-serine calculated from the alignment shown in *c* and plotted as a function of residue number. The dark grey curves represent the alignment on D1 lobes (left in *c*), the light grey curves the alignment on D2 lobes (right in *c*). The regions where these two curves intersect constitute the D1–D2 hinge region and are highlighted in pink. The diagram shows that the D1–D2 hinge consists of two parts, one in S1 (H_{S1}) and one in S2 (H_{S2}). The H_{S1} part is located at Tyr543, and we included the two residues around it for our experiments. The H_{S2} part is located around residues 760–768. As some residues in H_{S2} were not resolved in the *apo* or D-serine-bound structures, it was not possible to precisely determine the start of H_{S2}. However, as the lack of electron density in this region indicates dynamics in the structure, it is tempting to assume that these residues are part of an inter-domain hinge region. As Thr760 is conserved in all D-serine-binding iGluR subunits and the plots clearly indicate it to belong to lobe D2, it was not included in the hinge region. At the other end of H_{S2}, the two plots seem to intersect around Asp767, and Arg768 and Gly769 were also included. The two segments comprising the D1–D2 hinge region thus consist of: Asp542-Tyr543-Ser544 (H_{S1}) and Val761-Gly762-Asn763-Thr764-Val765-Ala766-Asp767-Arg768-Gly769 (H_{S2}).

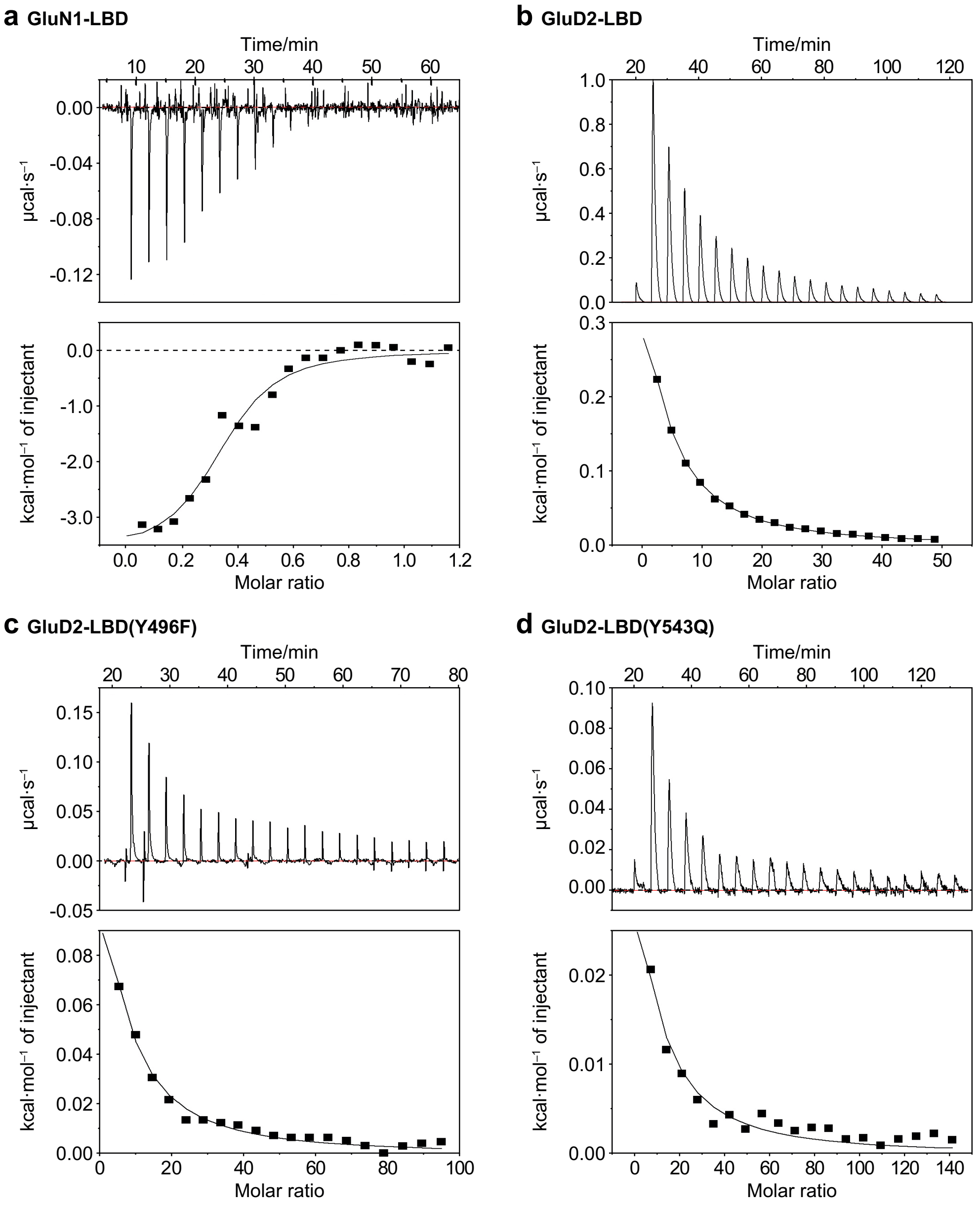
a GluN1-LBD
b GluD2-LBD
c GluD2-LBD(Y496F)
d GluD2-LBD(Y543Q)
Time/min
µcal·s⁻¹
kcal·mol⁻¹ of injectant
Molar ratio

Fig. S2 a-d

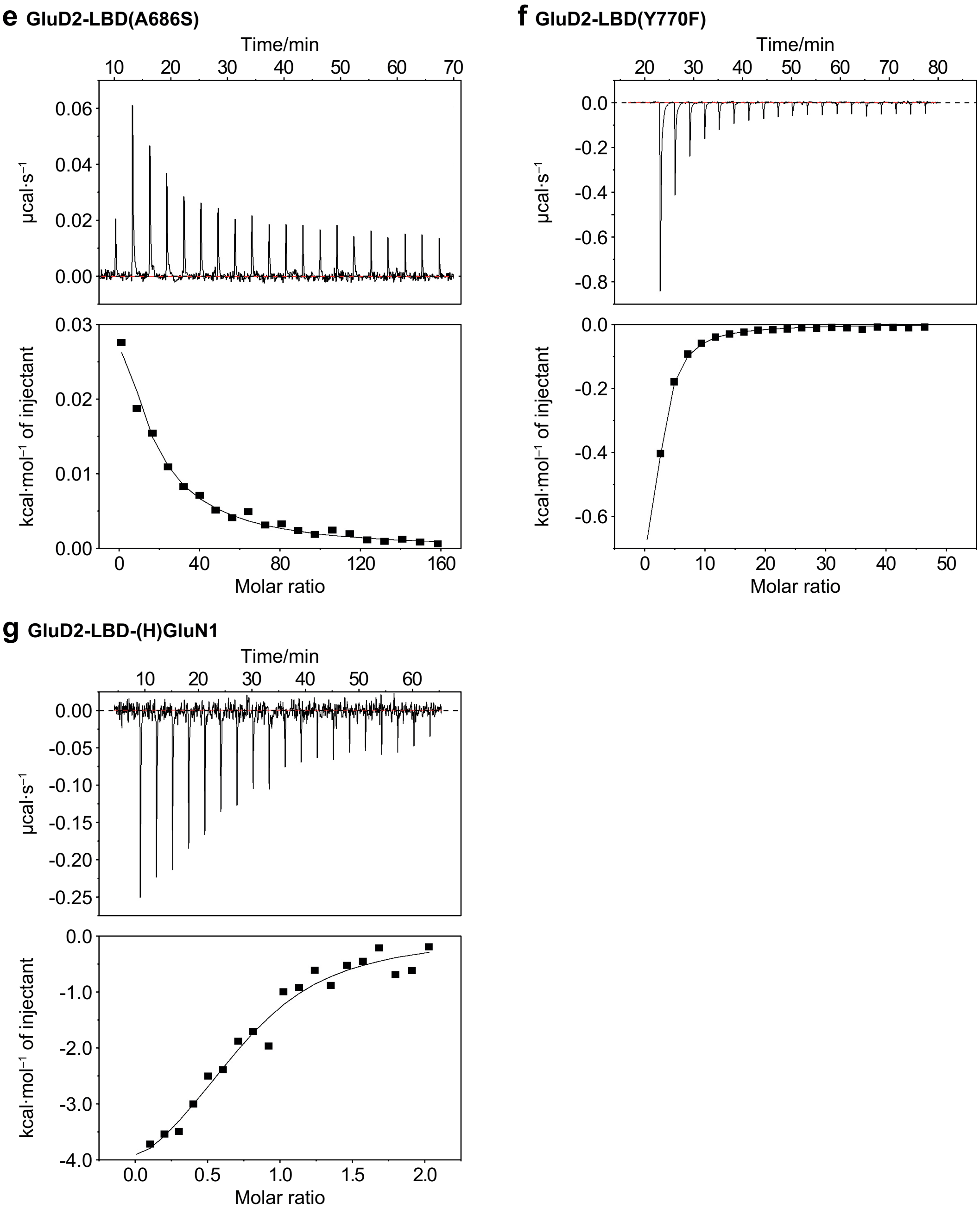

Fig. S2 Isothermal titration calorimetry data on binding of D-serine to wild-type GluN1-LBD (**a**), wild-type GluD2-LBD (**b**), binding site mutants (**c–f**), and GluD2-LBD-(H)GluN1 (**g**). Raw data are shown in the top panel and isotherms in the bottom panel. The data shown are representatives of three experiments. (**a**) GluN1-LBD at 20°C: Heat is developed after each injection (exothermic reaction, enthalpy-driven binding). (**b**) Wild-type GluD2-LBD at 25°C: Heat is absorbed after each injection (endothermic reaction, entropy-driven binding), the signal is diminished when the protein becomes saturated with D-serine. (**c**) GluD2-LBD(Y496F) at 25°C: Heat is absorbed after each injection (endothermic reaction, entropy-driven binding). (**d**) GluD2-LBD(Y543Q) at 25°C: Heat is absorbed after each injection (endothermic reaction, entropy-driven binding). (**e**) GluD2-LBD(A686S) at 25°C: Heat is absorbed after each injection (endothermic reaction, entropy-driven binding). (**f**) GluD2-LBD(Y770F) at 25°C: Heat is developed after each injection (exothermic reaction, enthalpy-driven binding). (**g**) GluD2-LBD-(H)GluN1 at 20°C: Heat is developed after each injection (exothermic reaction, enthalpy-driven binding).

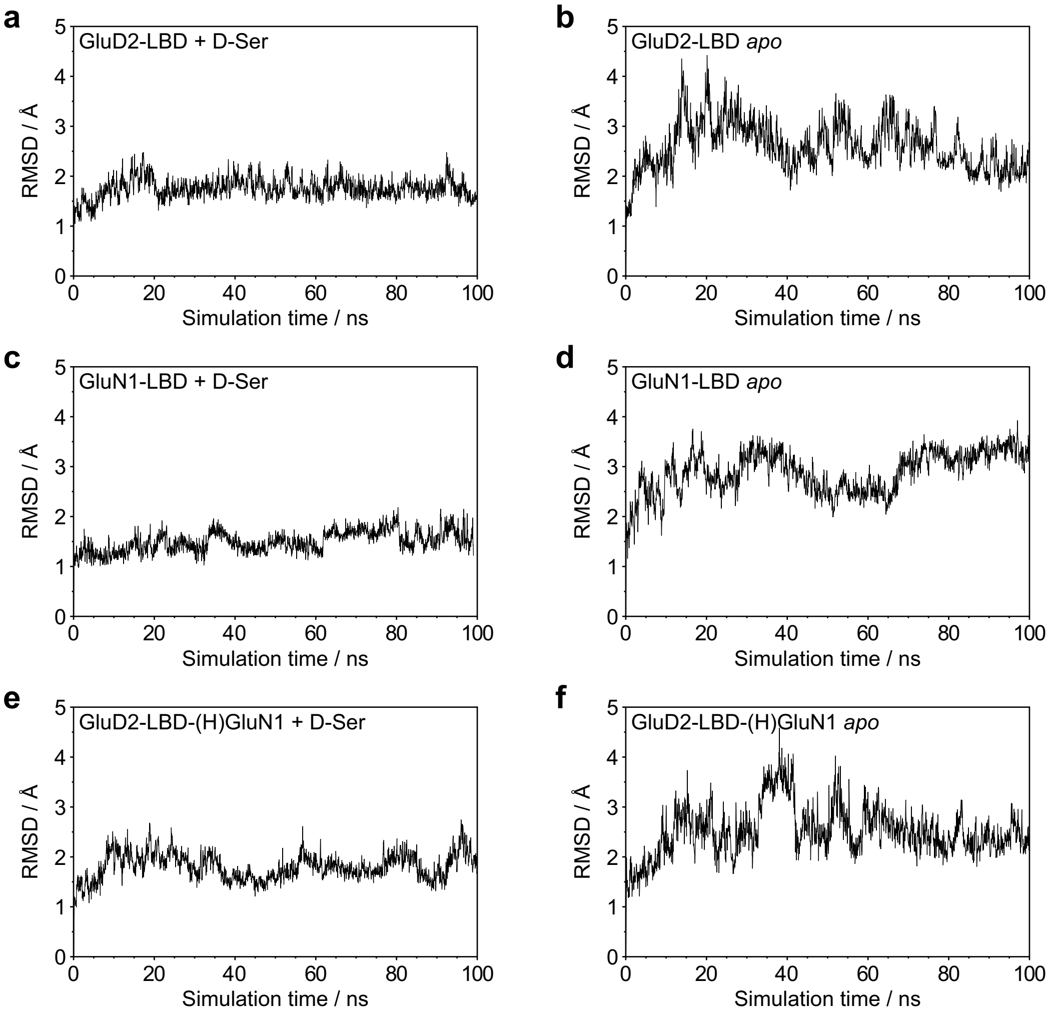

Fig. S3. RMSDs on all Cα atoms during the 100 ns MD simulations. (**a**) GluD2-LBD with D-serine. (**b**) GluD2-LBD *apo*. (**c**) GluN1-LBD with D-serine. (**d**) GluN1-LBD *apo*. (**e**) GluD2-LBD-(H)GluN1 with D-serine. (**f**) GluD2-LBD-(H)GluN1 *apo*.

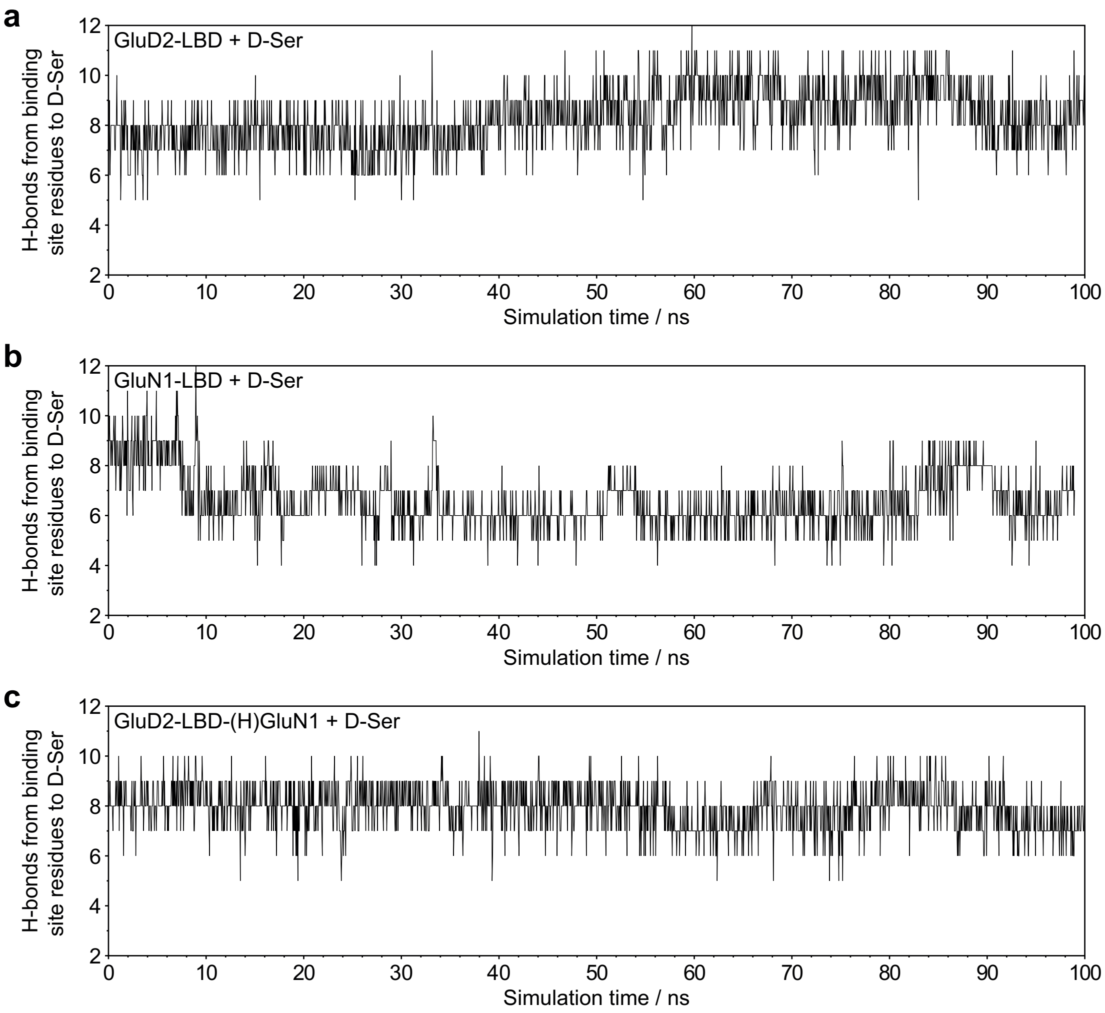

Fig. S4. Total number of hydrogen bonds from binding site residues to D-serine during the 100 ns MD simulations as calculated with GROMACS (gmx hbond). (**a**) GluD2-LBD with D-serine. (**b**) GluN1-LBD with D-serine. (**c**) GluD2-LBD-(H)GluN1 with D-serine.

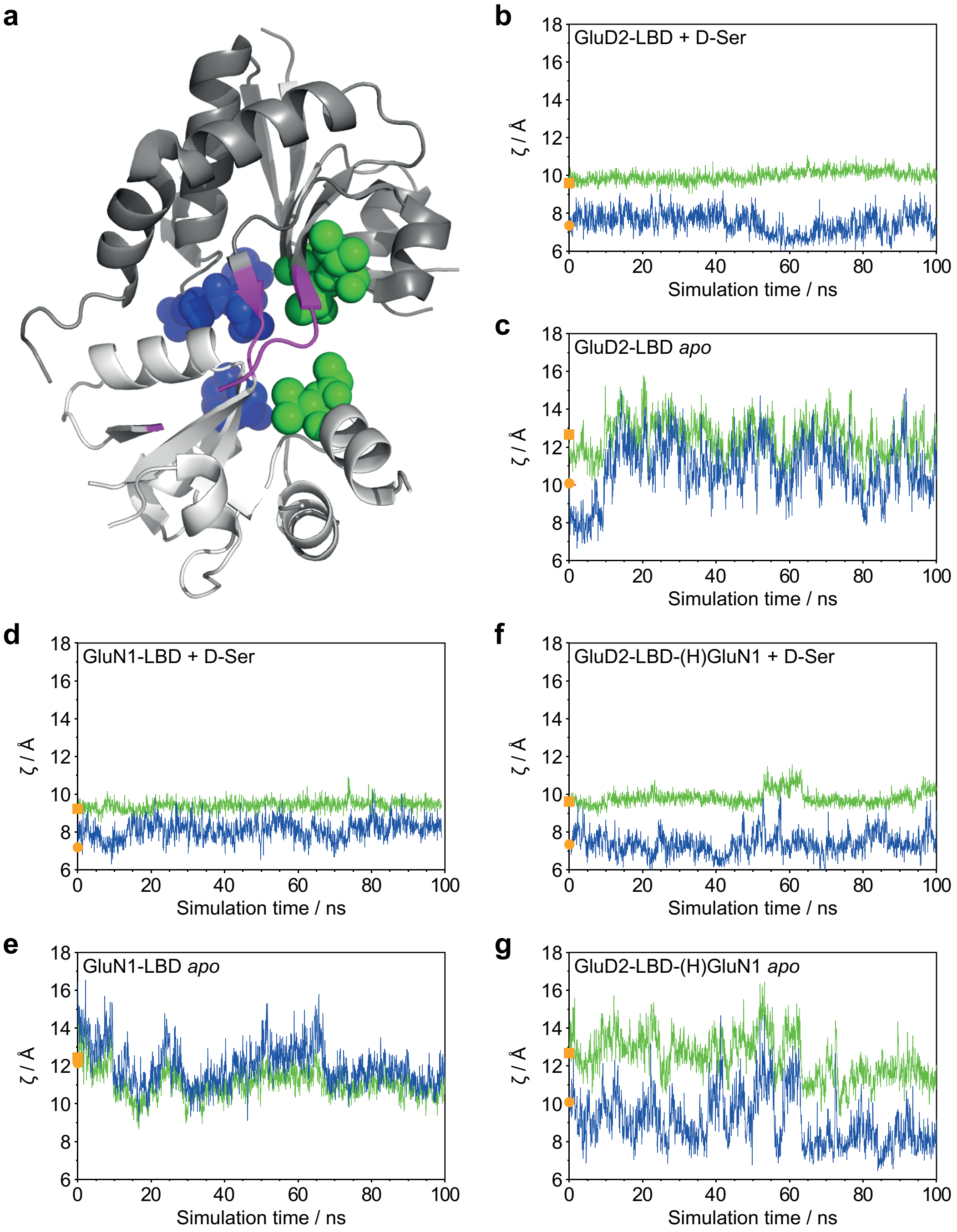

Fig. S5. ζ1 and ζ2 values during the 100 ns MD simulations.
(**a**) Cartoon representation of GluD2-LBD with definition of ζ1 and ζ2. Lobe D1 is shown in grey and lobe D2 in light grey. Residues used to define ζ1 (D1: Leu524, Thr525, Ile526 and D2: Ala686, Val687) are shown in blue and residues used to define ζ2 (D1: Leu449, Glu450, Glu451 and D2: Ser725, Gln726) in green. ζ1 and ζ2 are defined as the distance between the centers of masses (Lau, A.Y. & Roux, B. The hidden energetics of ligand binding and activation in a glutamate receptor. *Nat. Struct. Mol. Biol.* **18**, 283-287 (2011)). The filled orange circles and boxes correspond to the ζ1 and ζ2 values observed in the X-ray structures. (**b**) GluD2-LBD with D-serine. (**c**) GluD2-LBD *apo*. (**d**) GluN1-LBD with D-serine. (**e**) GluN1-LBD *apo*. (**f**) GluD2-LBD-(H)GluN1 with D-serine. (**g**) GluD2-LBD-(H)GluN1 *apo*.

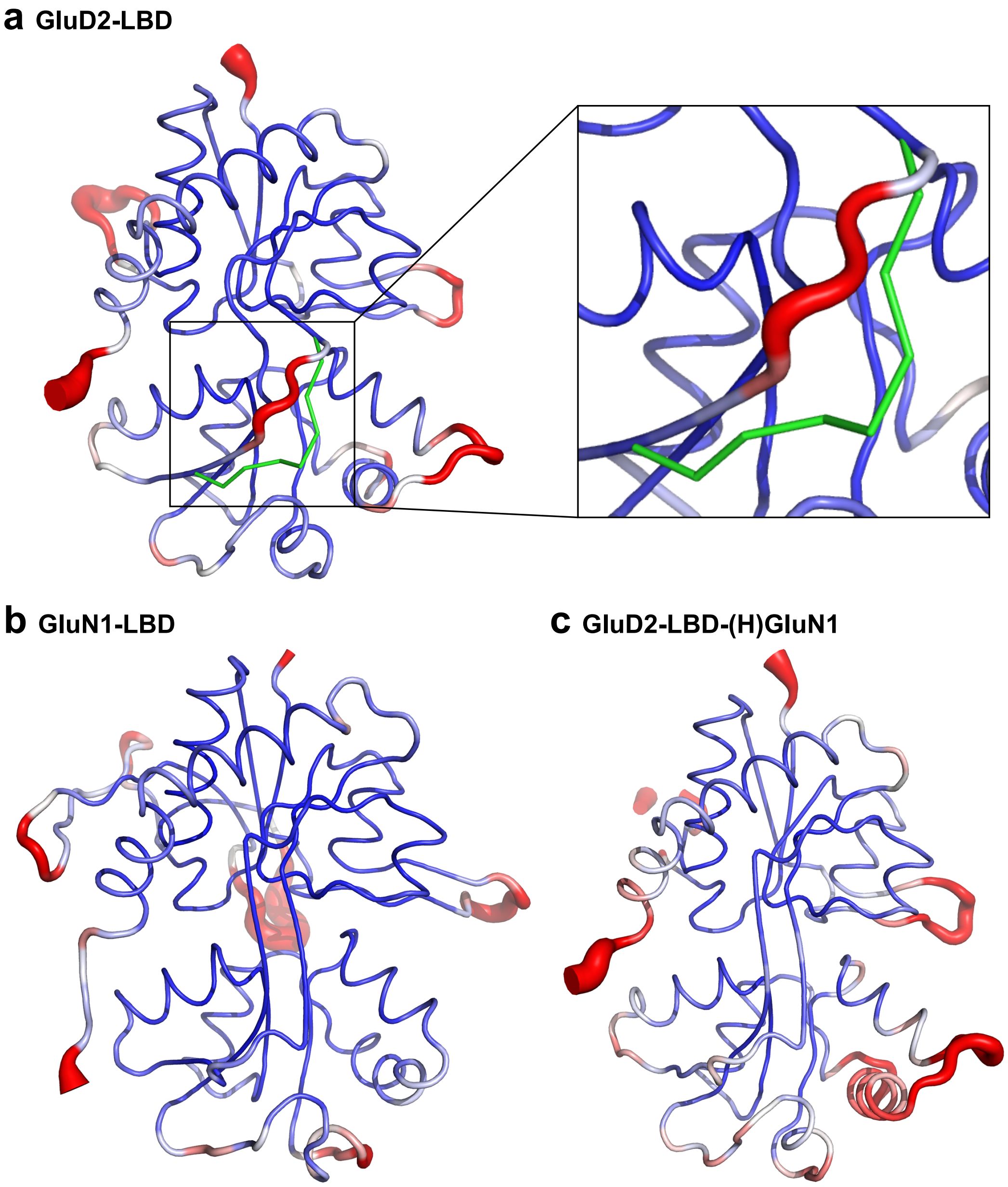

Fig. S6. Structures colored according to flexibility during 100 ns MD simulation. (**a**) Two major D1–D2 hinge region conformations are observed in GluD2-LBD with D-serine. One conformation of the GluD2-LBD structure is colored according to flexibility during the MD simulation (from blue – least flexible to red – most flexible). The second major hinge region conformation seen after 40 ns of simulation is shown in green. (**b**) GluN1-LBD with D-serine. (**c**) GluD2-LBD-(H)GluN1 with D-serine.

a **GluD2-LBD**

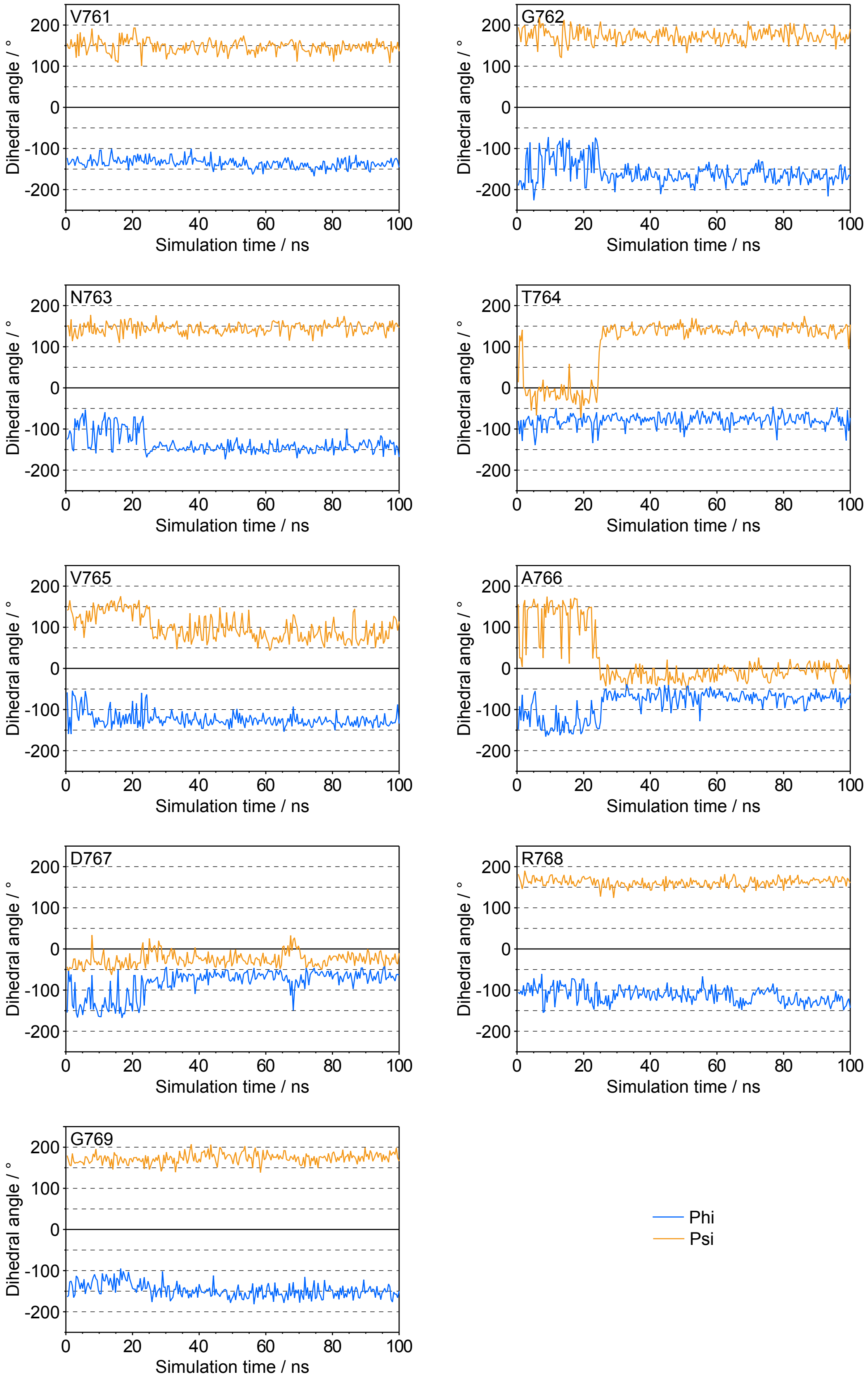

Fig. S7a

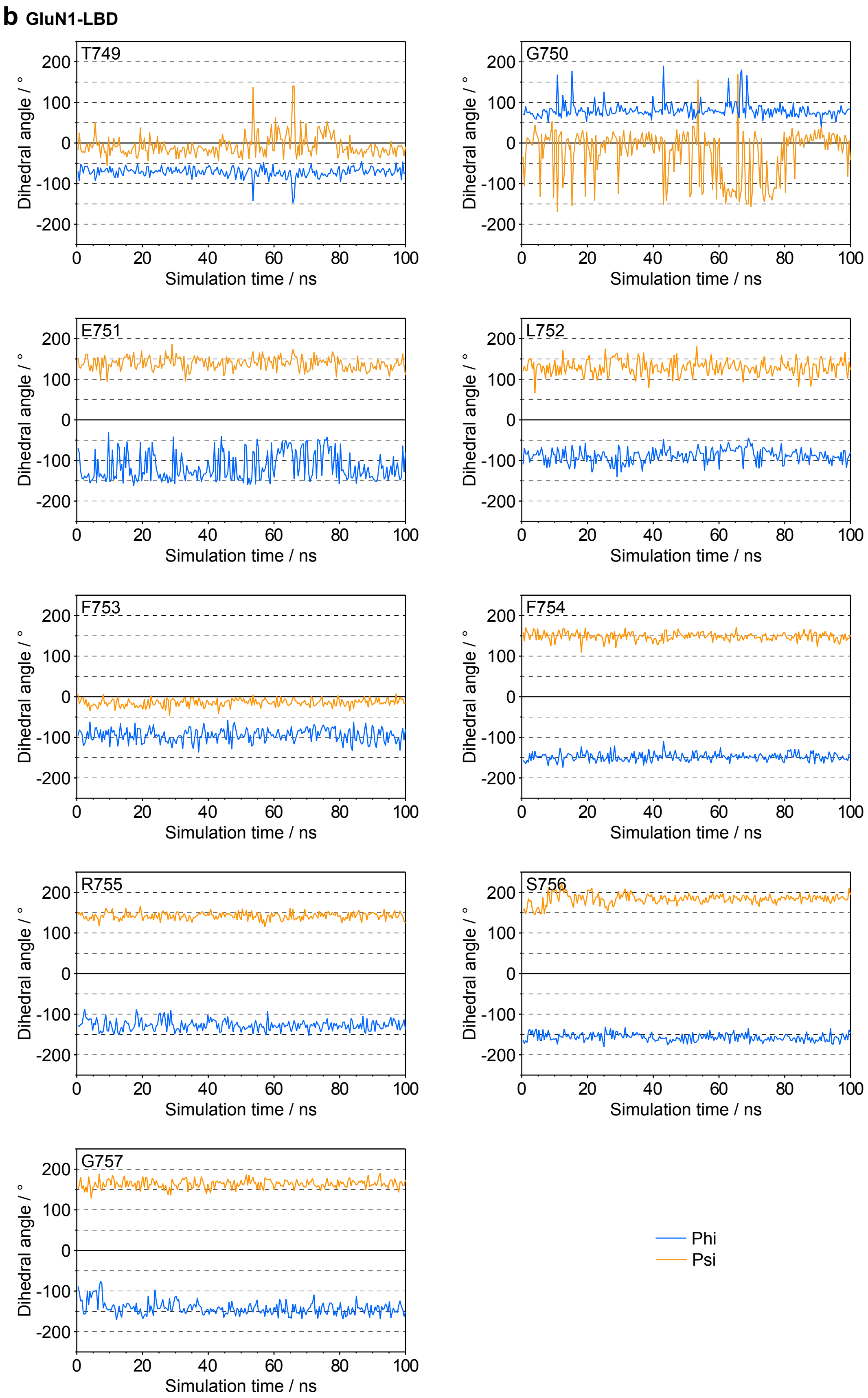
b GluN1-LBD
T749
G750
E751
L752
F753
F754
R755
S756
G757
Dihedral angle / °
Simulation time / ns
Phi
Psi

Fig. S7b

C **GluD2-LBD-(H)GluN1**

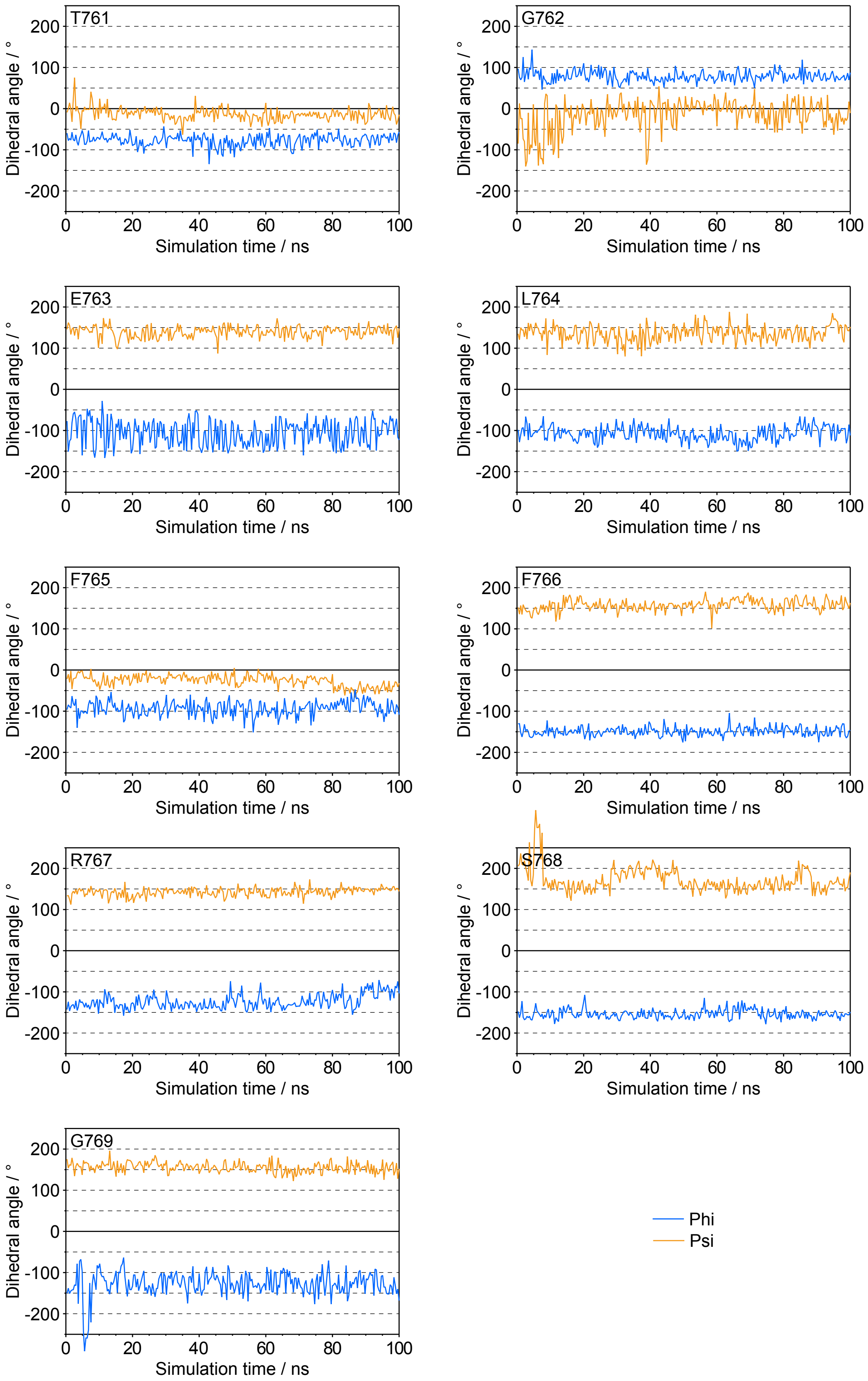

Fig. S7. Variation of the phi and psi torsional angles for the H_{S2} region during the 100 ns MD simulation. (**a**) GluD2-LBD with D-serine. (**b**) GluN1-LBD with D-serine. (**c**) GluD2-LBD-(H)GluN1) with D-serine.

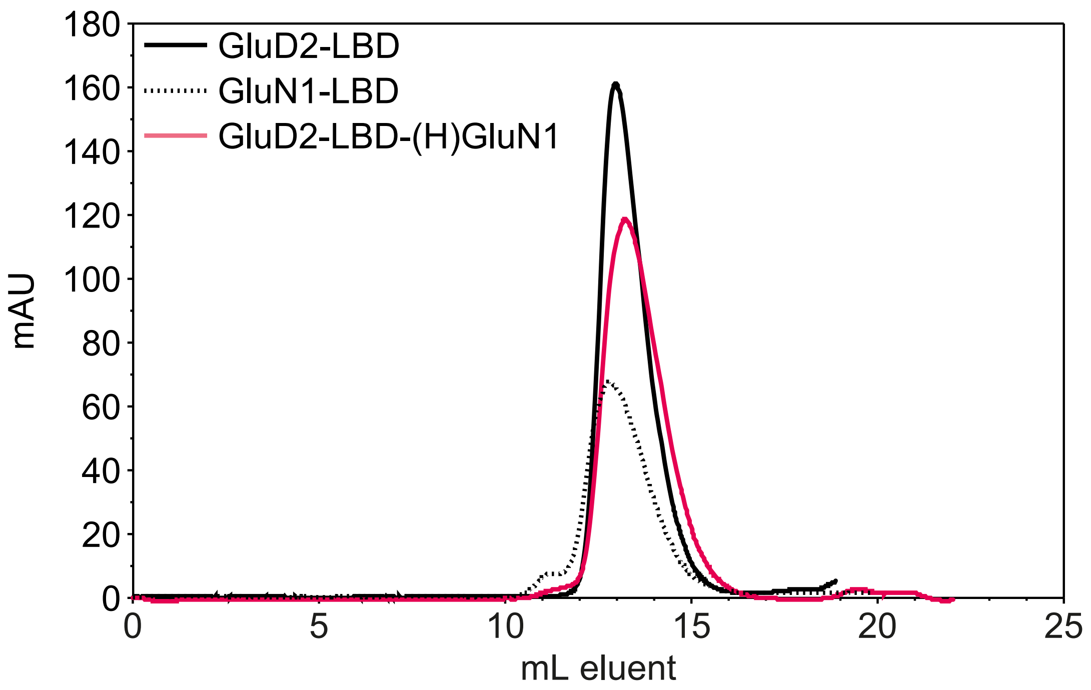

Fig. S8. Size-exclusion chromatography on GluD2-LBD (solid black), GluN1-LBD (dashed black), and GluD2-LBD-(H)GluN1 (pink). The peak volumes are 12.9 mL for GluD2-LBD, 12.8 mL for GluN1-LBD, and 13.2 mL for GluD2-LBD-(H)GluN1.

Table S1. Hydrogen bonding between D-serine and binding site residues during the 100 ns MD simulations. In column headings GluD2 numbering is shown without parentheses and GluN1 numbering in parentheses.

**Ligand-receptor contacts**			Tyr496 (Phe484)	Ala523 (Pro516)	Thr525 (Thr518)	Arg530 (Arg523)	Tyr543 (Gln536)	Ala686 (Ser688)	Asp742 (Asp732)
D-Ser	$-COO^-$	GluD2			NH, OH	guanidinium		NH	
D-Ser	$-COO^-$	GluN1			NH, OH	guanidinium		NH, OH	
D-Ser	$-COO^-$	GluD2-LBD-(H)GluN1			NH, OH	guanidinium		NH	
D-Ser	$-NH_3^+$	GluD2	OH	C=O	OH				COO^-
D-Ser	$-NH_3^+$	GluN1		C=O	OH				COO^-
D-Ser	$-NH_3^+$	GluD2-LBD-(H)GluN1		C=O	OH				COO^-
D-Ser	-OH	GluD2					OH		COO^-
D-Ser	-OH	GluN1			OH			NH, OH	COO^-
D-Ser	-OH	GluD2-LBD-(H)GluN1						NH	COO^-
